# These are the days of lasers in the jungle

**DOI:** 10.1186/s13021-014-0007-0

**Published:** 2014-09-03

**Authors:** Joseph Mascaro, Gregory P Asner, Stuart Davies, Alex Dehgan, Sassan Saatchi

**Affiliations:** 1 American Association for the Advancement of Science, 1300 New York Ave NW, Washington 20001, DC, USA; 2 Department of Global Ecology, Carnegie Institution for Science, 260 Panama St., Stanford 94305, CA, USA; 3 Forest Global Earth Observatory, Center for Tropical Forest Science, Smithsonian Tropical Research Institute, Washington 20013, DC, USA; 4 Conservation X Labs, 2380 Champlain St. NW #203, Washington 20009, DC, USA; 5 Jet Propulsion Laboratory, California Institute of Technology, 4800 Oak Grove Drive, Pasadena 91109, CA, USA

## Abstract

For tropical forest carbon to be commoditized, a consistent, globally verifiable system for reporting and monitoring carbon stocks and emissions must be achieved. We call for a global airborne LiDAR campaign that will measure the 3-D structure of each hectare of forested (and formerly forested) land in the tropics. We believe such a database could be assembled for only 5% of funding already pledged to offset tropical forest carbon emissions.

In a precious 152 minutes on the Lunar surface, Neil Armstrong and Buzz Aldrin raced to collect rocks, dust and photographs that are an enduring fascination in museums and laboratories—testament to the awesome effort expended to touch them. They left behind a discarded landing assembly, sprawling trails of footprints, and the flag of the United States. But they also left behind a science experiment about the size of a coffee table. This hunk of honeycombed metal may be the most important scientific legacy of the Apollo Program. It’s called a laser retroreflector, and it revolutionized our understanding of the Moon [1].

Laser ranging technology is straightforward: it works like radar or sonar but with lasers. Point a laser at a target and collect the reflected light bouncing back; with a stop watch fit for Einstein, you can measure the time delay between the laser shot and the return bounce to determine the distance to your target.

Since the experiment began, we’ve learned that the Moon’s orbit is widening by 3.8 centimeters per year, slowing the rotation of the Earth by about 2.3 seconds per century. These changes give us the leap second, and in a few million years will make February 29^th^ obsolete. The revelation has also dramatically influenced our thinking about the formation of the Earth-Moon system some 4.5 billion years ago.

Just as the Moon’s history was disrobed by laser ranging 50 years ago, Earth’s tropical forests are giving up their secrets to the light. Airborne light detection and ranging—called LiDAR—has over the last ten years become a key tool that ecologists use to understand physical variation in tropical forests across space and time [2],[3]. Like an MRI of the human brain, LiDAR probes the intricate three-dimensional architecture of the forest canopy, unveiling carbon that forests keep out of the atmosphere, and also the mounting threats to that carbon storehouse: drought, fire, clandestine logging and brash gold-mining operations [4]. Even the quintessential natural disturbance of the sun-filled light gap—long thought to enhance the incredibly high species diversity of tropical forests—has been deconstructed by laser technology [5].

Laser ranging in tropical forests is such a game-changing technology that science results can scarcely get through peer-review before they are dwarfed by still larger-scale studies. In a decade, laser power on commercial-grade LiDARs has skyrocketed and costs have plummeted. These improvements in LiDAR technology allow airplanes to fly faster, higher and farther, covering more forest area in a single day than every ground-based survey that has ever been collected in the history of tropical ecology. To estimate the amount of carbon stored in a 50-hectare tropical forest monitoring plot on the ground—the largest field plot in the world—takes a team of 12 people about eight months: a slog of rain and mud and snakes with tape measures and data log books [6]. Today’s airborne LiDARs can get you to within about 10% [7],[8] of the same carbon estimate in *eight seconds*.

It is this staggering contrast in scale between LiDAR and fieldwork that led us here: Before this decade is out, we could directly assess the carbon stock of every single square hectare of tropical forest on Earth. We could do it just as well as if we were standing there in the flesh with tape measures in hand. And we could do it for far less than what we have already spent to offset carbon emissions from forests.

## “A loose affiliation of millionaires and billionaires”

The United Nations Framework Convention on Climate Change is the international venue in which tropical forest carbon accounting programs and practices have been negotiated, the most visible of these being REDD + (Reduced Emissions from Deforestation and Degradation). When first imagined, the precursor of REDD + was straightforward: landowners and governments would draw revenue from a global carbon market if they could verifiably reduce their forest-sector carbon emissions to the atmosphere. The policy has at times seemed like a real possibility, but has recently faced major setbacks.

At present, more than a dozen countries are participating in preliminary work to develop REDD + implementation strategies. These efforts involve the building of infrastructure and expertise, including consulting and contracting with various academic groups and non-government organizations to estimate carbon stocks and emissions. Pledged REDD + funding now exceeds $5 billion [9]. These funds come partly from donors but primarily from governments.

Although the UNFCCC has outlined general guidelines for implementing a monitoring, reporting and verification program, most REDD + projects remain in the demonstration phase, and thus the funds are being spent in myriad ways. Because of the urgency in developing the program to meet scattershot international deadlines and twisting geopolitical expectations—current efforts to assess tropical forest carbon are highly inconsistent.

But for REDD + to function, a consistent, globally verifiable system for reporting and monitoring carbon stocks and emissions in tropical forests must be achieved in the near future. Without such an accounting system, the amount of avoided carbon emissions from deforestation and degradation achieved would not be quantifiable and thus the program would not be tractable.

The time has come for a brute-force effort to directly assess the carbon stock for all of the world’s tropical forests by 2020. Airborne LiDAR is uniquely suited for this role because it can be collected, standardized, reported and verified in a simple manner by both a landholder and any third party.

We call for a global airborne LiDAR campaign that will measure the 3-D structure of each hectare of the Earth’s non-barren land surface between the Tropic of Capricorn and Tropic of Cancer. Our targets are approximately 1,500,000,000 individual hectares of the richest and fastest disappearing habitats in the world, and the human-used lands that have replaced them.

## “Staccato signals of constant information”

It is easy in principle, though logistically nightmarish, to measure carbon in tropical forests. A strict constructionist would cut, dry and weigh the biomass of the world’s forests. But this is a self-defeating enterprise. As a result, it is likely that no one has measured carbon over a single hectare of tropical forest, even with the most detailed field surveys. For a century ecologists and foresters have relied on allometric estimation in lieu of carbon measurements to translate field surveys of tree diameters, heights and wood densities into whole-forest carbon estimates. Given a volume with known dimensions and density, one would estimate its mass in a similar fashion.

As the new kid on the block, LiDAR has been tacked onto the back end—initially thought of as kind of large-scale helper to field surveys. Carbon estimates from the field have been treated as something inherently closer to the real thing than measurements made by LiDAR—ground “Truth” with a capital “T”. This is perhaps understandable historically, but vis-à-vis actual carbon, there is no such thing as ground truth: both field and LiDAR efforts rely on allometry to convert measurements into carbon estimates [10]. *Prior* to using these measurements for carbon estimation, they exist as standardized, spatially explicit, archivable and verifiable data—the needed substrate for a REDD-type accounting program.

Due to the constancy of the underlying measurements, both field and LiDAR data could provide the needed information if they covered every hectare on Earth. But, in the case of field surveys, this is impossible. The surveys that do exist measure a tiny amount of actual forest, and so what might be verified is widely spaced. And to avoid fraud and protect landowners, many governments keep their plot locations secret. Satellite LiDAR data remain sparse, providing only extrapolated, coarse-resolution carbon estimates with very high uncertainties [11], and there is no prospect of wall-to-wall coverage in the near future [12]. By 2020, airborne LiDAR could give us a direct measurement of 3-D forest structure for every hectare in the tropics: a standardized database from which to build a carbon economy.

## “Don’t cry, baby, don’t cry”

The 1993 film *Jurassic Park* is filled with brilliant insights into science, from Malcolm’s cautionary tale on the wisdom of cloning to Sattler’s diatribe about human control over nature. The best of these is professor Alan Grant’s reaction to a sonar return of a still-entombed velociraptor skeleton. The lowly grad student showing him the return quips: “a few more years and we won’t have to dig anymore”. He replies, dismayed: “Where’s the fun in that?”

Ecologists often believe that embracing LiDAR technology will somehow mean the end of fieldwork. It won’t. LiDAR has initially enhanced the role of fieldwork: even though many plots were established before carbon assessment became a key objective in tropical forest science, these plots are a critically important resource for understanding how LiDAR 3-D measurements can be used to estimate carbon stocks. Very soon, plot-based harvests will allow remote sensing data to relate directly to measurements of carbon stocks in closed canopy forests—something that has recently been accomplished in a tropical savanna [13]. And beyond carbon applications, LiDAR data are revolutionizing our understanding of basic tropical forest ecology, from environmental controls to succession e.g., [5],[14],[15].

The other reason to smile? The price tag. Our ambitious plan can be accomplished for far less than what we have already spent on avoided deforestation. Aircraft leasing, data collection and processing costs for 30 days of flying can reasonably be limited to $500,000 [16]. Using this monthly sampling unit, collecting at an average of 100,000 hectares per day, a fleet of ten aircraft could do the job in four years at US$250 million, or just 5% of pledged REDD + funding.

At that rate, we can lose a year and a half to torrential rain and still get the job done.
